# Supplementary figures and images for: Engineering Rapalog-Inducible Genetic Switches Based on Split-T7 Polymerase to Regulate Oncolytic Virus-Driven Production of Tumour-Localized IL-12 for Anti-Cancer Immunotherapy
Source: Pharmaceuticals (Basel). 2023 May 7;16(5):709. doi: 10.3390/ph16050709 (PMC10224353; doi:10.3390/ph16050709)

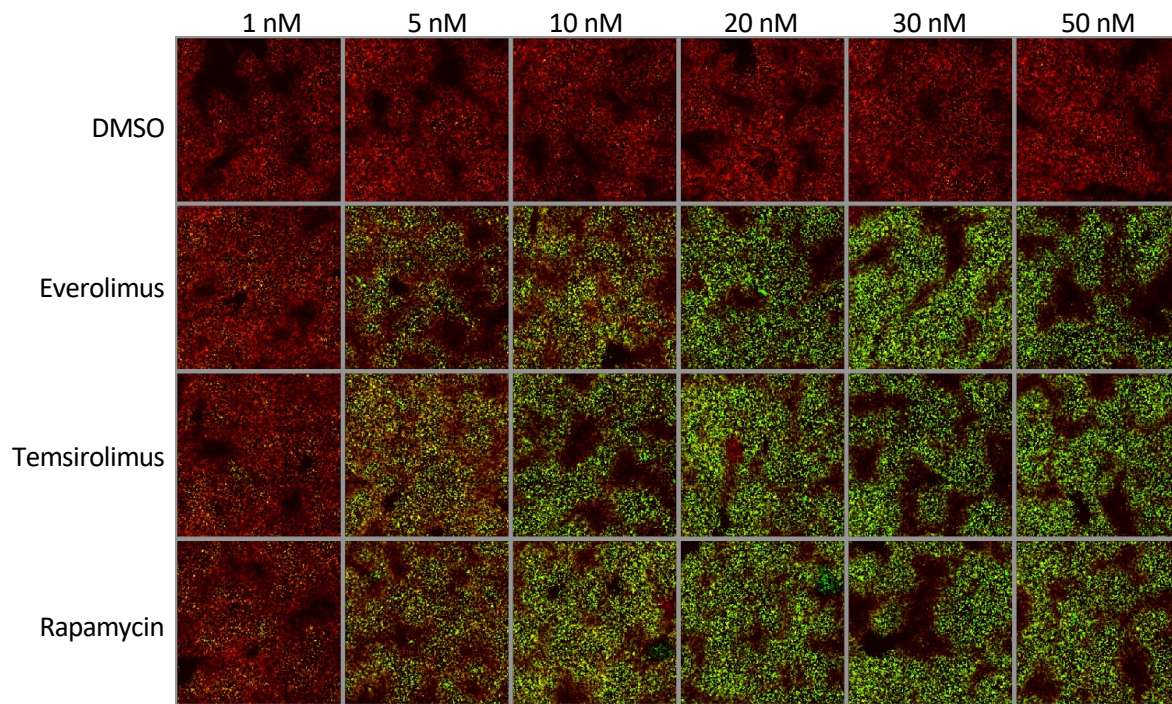**b**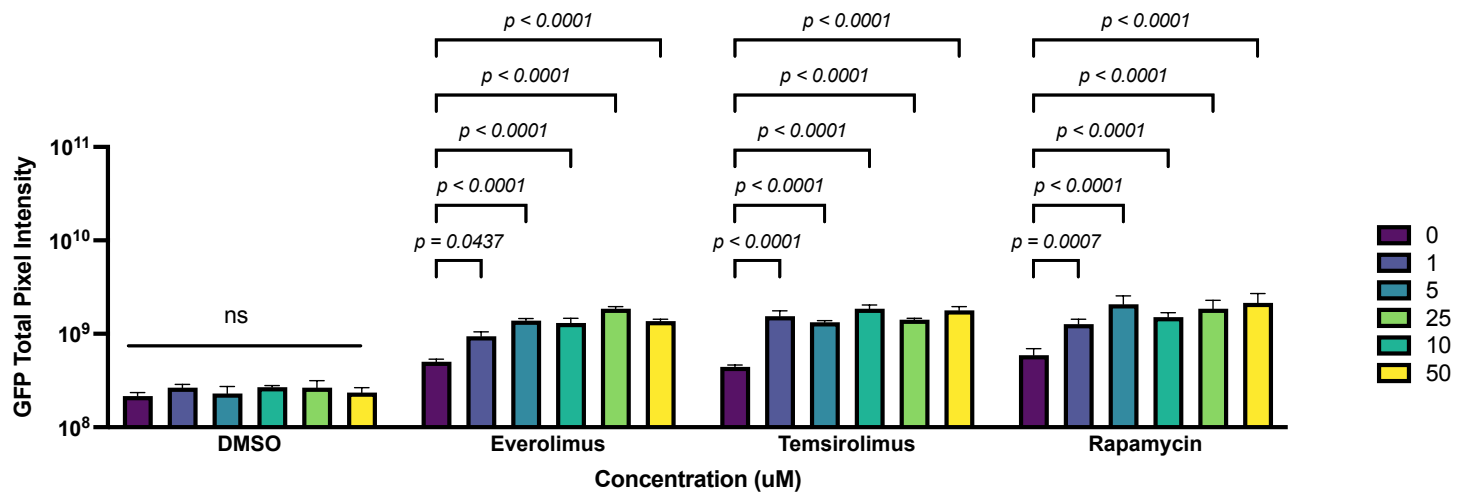

**C**

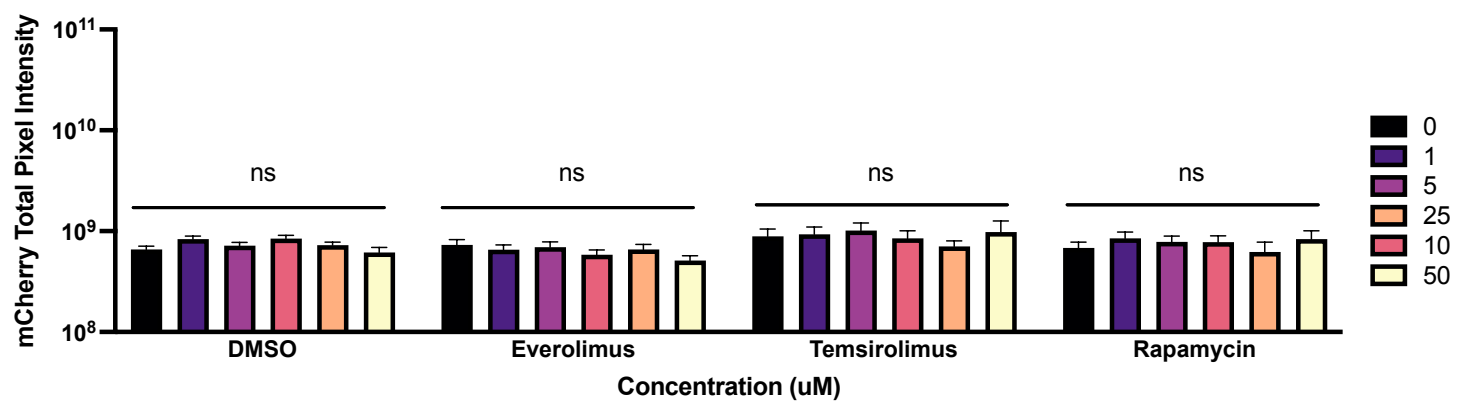

**a**

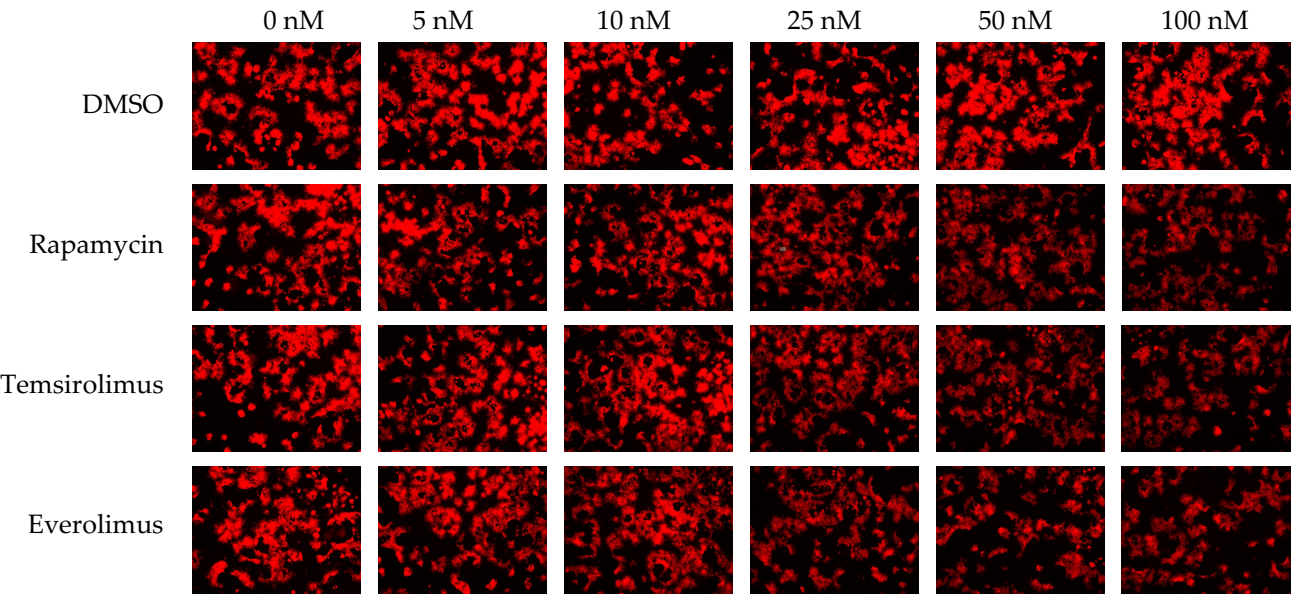

**b**

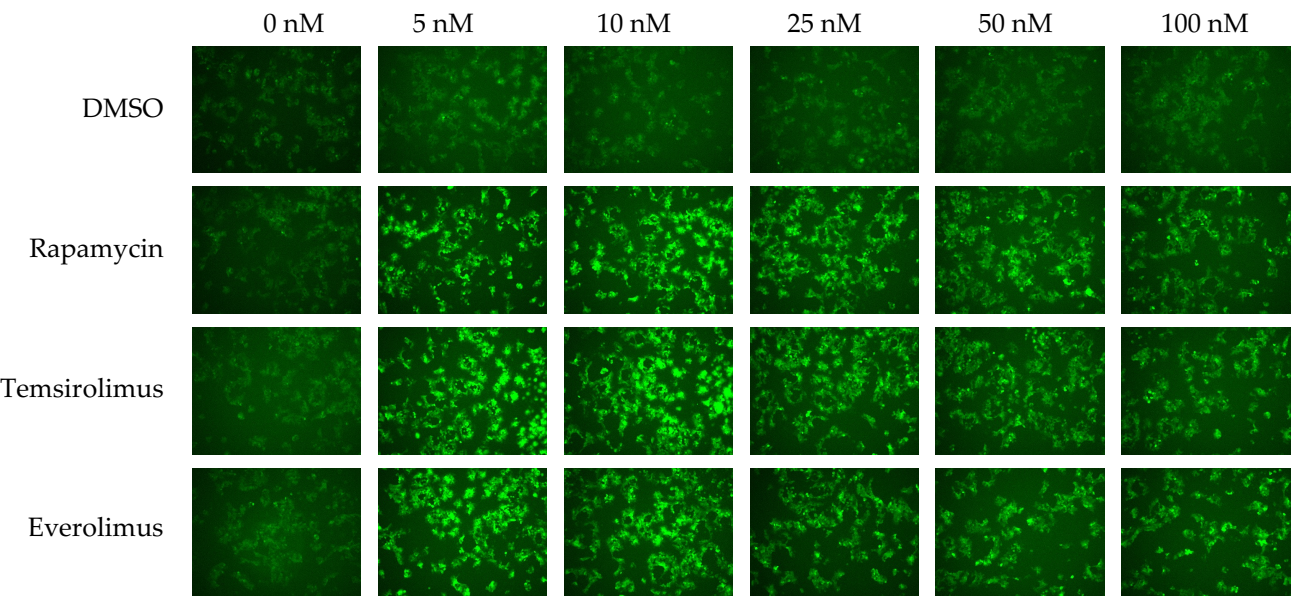

a

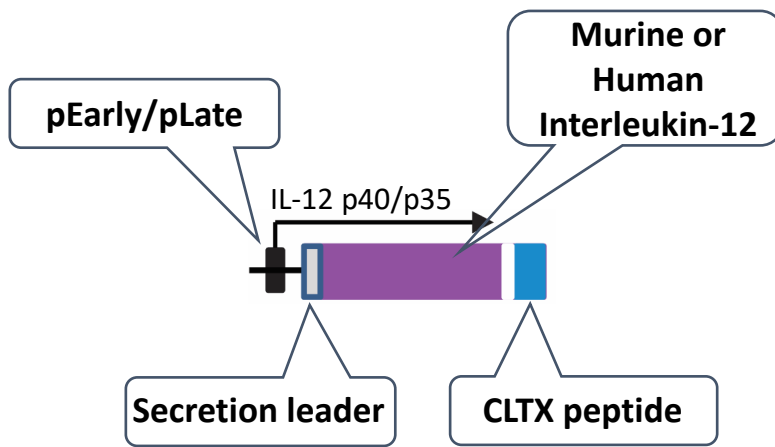

b

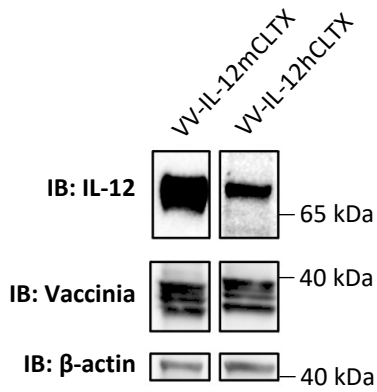

c

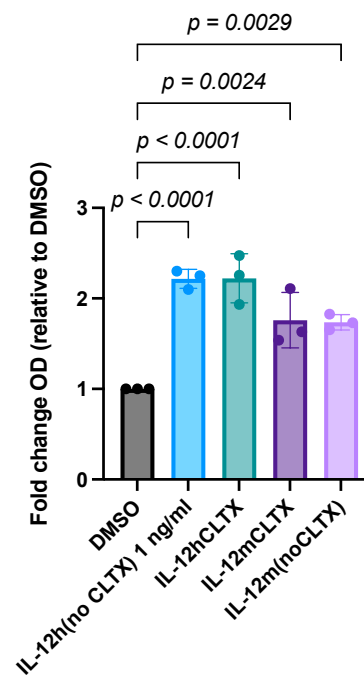

a

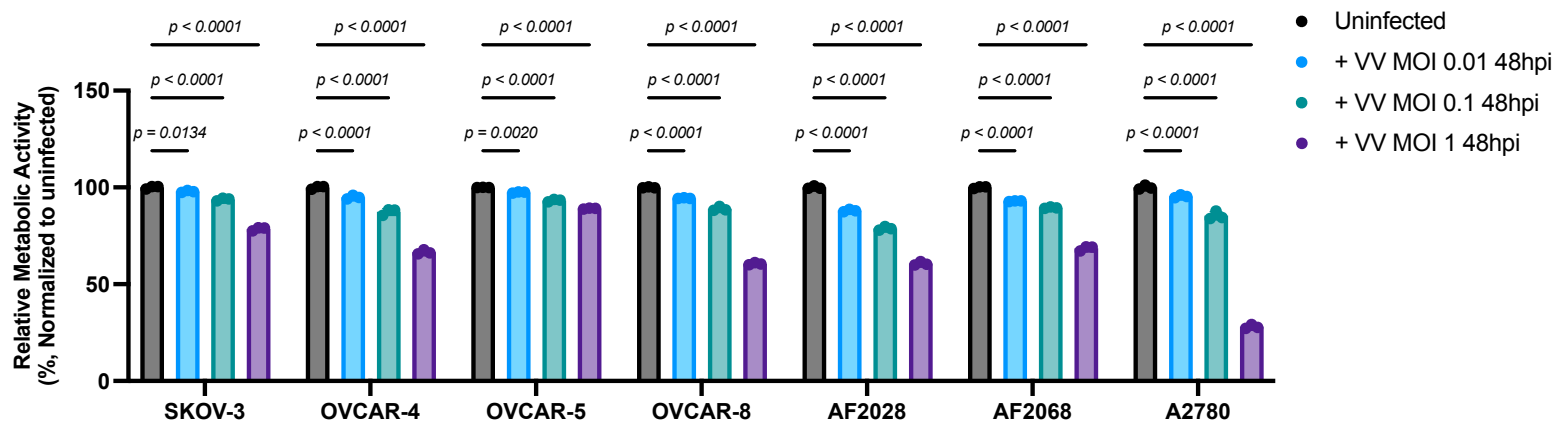

b

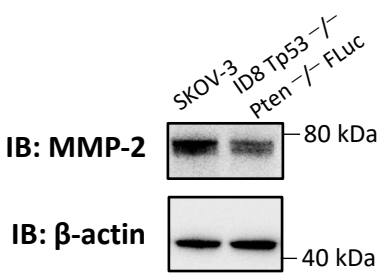

c

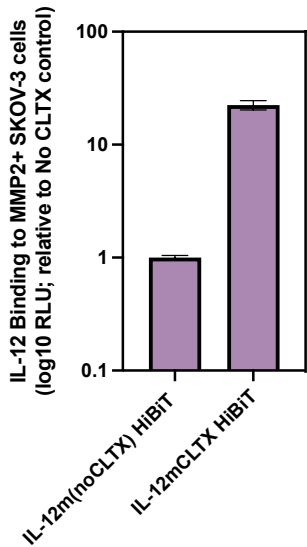

d

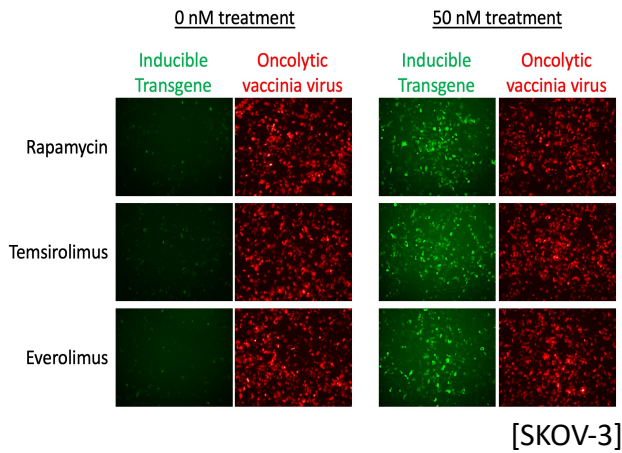

e

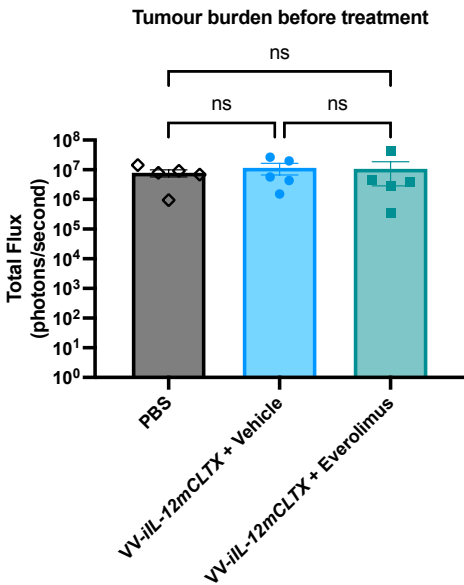

Supplement: Supplementary file 1 [file pharmaceuticals-16-00709-s001.zip › pharmaceuticals-2301792-supplementary.pdf]
